# Analysis of Electrochemically Active Substances in Malvaceae Leaves via Electroanalytical Sensing Technology for Species Identification

**DOI:** 10.3390/mi14020248

**Published:** 2023-01-18

**Authors:** Qiong Wang, Weiting Ye, Dongling Li, Jiangwei Zhu, Chenghang Liu, Chengte Lin, Li Fu, Zenglai Xu

**Affiliations:** 1 Institute of Botany, Jiangsu Province and Chinese Academy of Sciences, Nanjing 210014, China; 2 Jiangsu Key Laboratory for the Research and Utilization of Plant Resources, Nanjing 210014, China; 3 College of Materials and Environmental Engineering, Hangzhou Dianzi University, Hangzhou 310018, China; 4 Co-Innovation Center for Sustainable Forestry in Southern China, Nanjing Forestry University, Nanjing 210037, China; 5 Key Laboratory of Marine Materials and Related Technologies, Zhejiang Key Laboratory of Marine Materials and Protective Technologies, Ningbo Institute of Materials Technology and Engineering (NIMTE), Chinese Academy of Sciences, Ningbo 315201, China

**Keywords:** electrochemical fingerprint, phytochemistry, electrochemical sensor, pattern recognition, phylogenetics

## Abstract

Electrochemical analysis has become a new method for plant analysis in recent years. It can not only collect signals of electrochemically active substances in plant tissues, but can also be used to identify plant species. At the same time, the signals of electrochemically active substances in plant tissues can also be used to investigate plant phylogeny. In this work, we collected electrochemical finger patterns in Malvaceae leaves based on the established methodological strategy. After the second derivative treatment, the collected electrochemical fingerprints can show more obvious differences. Three different recognition models were used to attempt electrochemical fingerprinting. The results show that linear support vector classification can be used to identify species with high accuracy by combining the electrochemical fingerprint signals collected in the phosphoric acid buffer solution and acetic acid buffer solution. In addition, the fingerprint information collected by the electrochemical sensor is further used for phylogenetic investigation. The 18 species were divided into three clusters. Species of the same genus have been clustered together. Dendrogram obtained by electrochemical fingerprinting was used to compare previously reported results deduced from morphological and complete chloroplast genomes.

## 1. Introduction

Malvaceae is the most evolved monophyletic group among the four core members of Malvales (the other three families are Sterculiaceae, Bombacaceae, and Tiliaceae) [1,2]. There are approximately 243 genera and more than 4200 species in the whole world, which are mostly distributed in tropical and subtropical regions, and also in temperate regions. There are more than 80 species in 20 genera in China, which have important economic uses, such as fiber raw material, medicinal, edible, and ornamental. There have been many controversies in the systematic study of Malvaceae. Edlin [3], Kearney [4], Bates [5], and Fryxell [6] divided each genus of Malvaceae into different groups and subgroups. As for the discussion on the phylogenetic relationship of Malveae members, most scholars believe that the they were divided into two branches in their early evolution [7,8], but there are still great disputes about which genera are included in each branch and their evolutionary relationship. On the other hand, in the discussion of the phylogenetic relationship among Malvaceae groups, different scholars have their own views on their phylogenetic positions [9,10,11]. In addition, the attribution of *Kydia* has been controversial, and Edlin [3], Fryxell [6], and Pfeil et al. [12] have all put forward different views. The systematic position of the genus *Abelmoschus* is also debated; Kearney [4], Fryxell et al. [13,14], Pfeil et al. [12], Koopnam Werner et al. [15], and Werner et al. [16] have discussed whether *Abelmoschus* should be classified into *Hibiscus* or be a separate genus through morphological and molecular biological studies. Recent molecular studies have shown that *Abelmoschus* is a monophyletic group originating from *Hibiscus* [16].

There are many techniques used to investigate plant phylogenetic positions, including morphological techniques, molecular techniques, and chemical composition techniques. Among them, chemotaxonomy is the technique of reflecting the relationship between plants by the differences in the chemical composition of their tissues. The similar chemical composition indicates that there are relatively similar genes among species, which further indicates that they have a close phylogenetic relationship. Chemical-based taxonomy has several advantages over molecular techniques, not least that it is faster and cheaper. Electrochemical fingerprinting in chemotaxonomy is a new technology that has been proposed in recent years. It shows the difference of kinship between different plants by the difference of electrochemically active substances in plant tissues. Our previous works have successfully confirmed the feasibility of this technique in phylogenetic investigations [17,18,19,20,21,22,23,24]. The electrochemical active components of plants, such as flavonoids and phenols, fluctuate according to species’ distance from each other.

Image-based plant recognition technology has been widely commercialized [25], and it performs very well in commercial plants and flowers. This is because sufficient pictures of plants were used for training. This is something that electrochemical fingerprint technology at the present stage cannot achieve. However, plant recognition based on image technology still faces challenges in the recognition of some species with similar morphological characteristics [26]. In particular, some species are easily recognized during some growth periods (e.g., flowering, fruiting), but exhibit very similar morphology characteristics during others. On the other hand, the accuracy of plant recognition technology based on image is not ideal in the identification of non-commercial plants [27]. This is because not enough images of these species have been used for training because they have only received attention from certain research groups. The identification technology based on electrochemical fingerprinting can complement the traditional plant identification technology. Rapid identification of species in a small range can be established by rapid fingerprint collection.

In this work, we further used electrochemical fingerprinting technology to collect the fingerprints of the electrochemically active substances in Malvaceae species. In addition to the conventional fingerprint analysis, this work is the first to optimize the range of data used for species identification. This also reduces the impact of background signals on the occurrence of investigations in the phylogenetics.

## 2. Materials and Methods

Leaves of *Abelmoschus esculentus*, *Abelmoschus manihot*, *Abelmoschus sagittifolius*, *Abutilon theophrasti*, *Alcea rosea*, *Hibiscus hamabo*, *Hibiscus moscheutos*, *Hibiscus mutabilis*, *Hibiscus sabdariffa*, *Hibiscus sinosyriacus*, *Hibiscus syriacus*, *Hibiscus syriacus* f. *albus-plenus*, *Hibiscus trionum*, *Kosteletyzkya virginica*, *Pentapetes phoenicea*, and *Urena lobata* were collected from Nanjing Botanical Garden, Memorial Sun Yat-Sen. Only mature and healthy leaves were harvested. All samples were kept frozen (−20 °C) before analysis.

Detail of the parameters of extraction preparation and electrochemical fingerprints collection are described in detail in the supporting materials (Supporting Information S1 and S2).

The second derivative is used for electrochemical fingerprinting of all species. Stoichiometric methods and machine learning algorithms were used to identify differences in the electrochemical fingerprint data of different species between samples. Partial least square-discriminant analysis (PLS-DA), linear support vector classification (LinearSVC), and random forest (RF) were used to identify different species. The confusion matrix is used to evaluate the effectiveness of the classification model.

## 3. Results and Discussion

**Figure 1:**
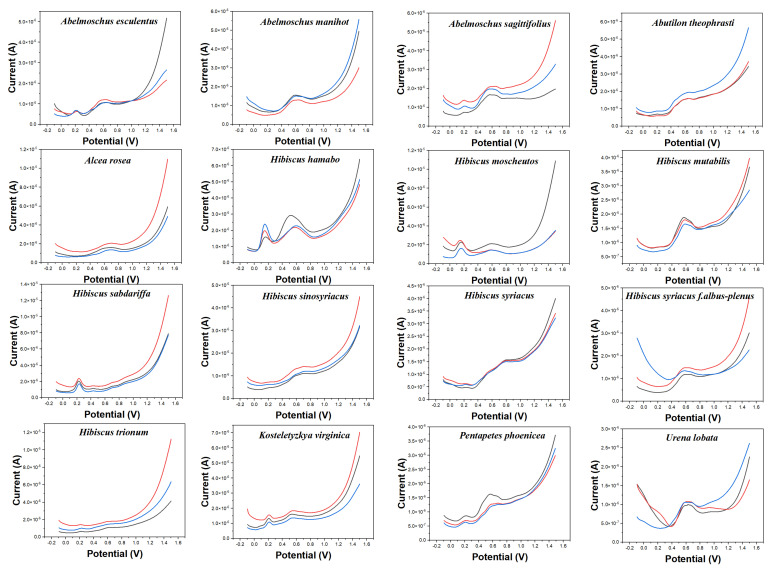
Electrochemical fingerprints of *Abelmoschus esculentus*, *Abelmoschus manihot*, *Abelmoschus sagittifolius*, *Abutilon theophrasti*, *Alcea rosea*, *Hibiscus hamabo*, *Hibiscus moscheutos*, *Hibiscus mutabilis*, *Hibiscus sabdariffa*, *Hibiscus sinosyriacus*, *Hibiscus syriacus*, *Hibiscus syriacus* f. *albus−plenus*, *Hibiscus trionum*, *Kosteletyzkya virginica*, *Pentapetes phoenicea*, and *Urena lobata*, recorded after water extraction under 0.1 M PBS (pH 7.0).

Electrochemical fingerprint collection is used to record the oxidation-reduction signals of electrochemically active molecules in plant tissues. At present, electrochemical voltammetry is mostly used in the determination of single small molecules with excellent properties, such as ketones [28], aldehydes [29], sugars [30], etc. These electrochemically active substances are widely found in plant tissues [31]. In contrast, electrochemical fingerprinting technology is used to collect the signals of all the electrochemically active substances in a complex system. Plant species are identified by differences in the signals of these substances in electrochemical reactions. Figure 1 shows the electrochemical behavior of the leaves of 16 species collected in this work after extraction (water as solvent) in 0.1 M PBS. It can be seen that different species exhibit different electrochemical behaviors. The background of the electrode in PBS is shown in Figure S1. It can be seen that the glassy carbon electrode increases of current over 1.0 V, but there is no obvious electrochemical oxidation peak. Although the DPV curves of three independent samples (from three individual plants) of the same species do not coincide exactly, they all exhibit consistent trends and characteristics. This represents the consistency of the chemical composition of the same species [32]. This is because the chemical composition of different species is regulated by genes [33]. However, the levels of these chemicals can vary depending on factors such as soil, sunlight, and moisture. This phenomenon has been confirmed by many research on phytochemistry [34,35,36]. Therefore, although their electrochemical behavior exhibits almost similar characteristics, they are not uniform in the current value. The oxidation behavior produced by these electrochemical fingerprints can be attributed to the oxidation of a series of electrochemically active molecules in plant tissues, such as phenolic compounds and aldehyde compounds. For example, Liu et al. [37] reported that luteolin can oxidase on a glassy carbon electrode surface at 0.4 V at a similar condition. Hendrickson et al. [38] reported the electrochemical behaviors of a series of catechol-containing flavonoids under PBS (pH 7.0) using a glassy carbon electrode. Their oxidation peaks were all in the range of 0–1.1 V. Luo and Liu [39] reported the electrochemical oxidation of vanillin around 0.6 V on a glassy carbon electrode surface. Our previous study also investigated the electrochemical oxidation of vanillin on a glassy carbon electrode surface [40].

The electrochemical behavior of some of these species shows some similarities. For example, *Abelmoschus esculentus* and *Abelmoschus sagittifolius* both show a smaller oxidation peak around 0.20 V. At the same time, they all exhibit a significant oxidation peak around 0.60 V. However, the oxidation peak of *Abelmoschus sagittifolius* at around 0.60 V is a double peak, while *Abelmoschus esculentus* is a rounded single peak. *Hibiscus mutabilis* also exhibits a very similar oxidation peak at around 0.60 V, but it does not oxidize substances at around 0.20 V. Similarly, the electrochemical fingerprints of *Alcea rosea* and *Hibiscus sinosyriacus* show only a gentle oxidation peak. Although they differ in slope at the oxidation starting potential, these differences are difficult to describe visually.

**Figure 2:**
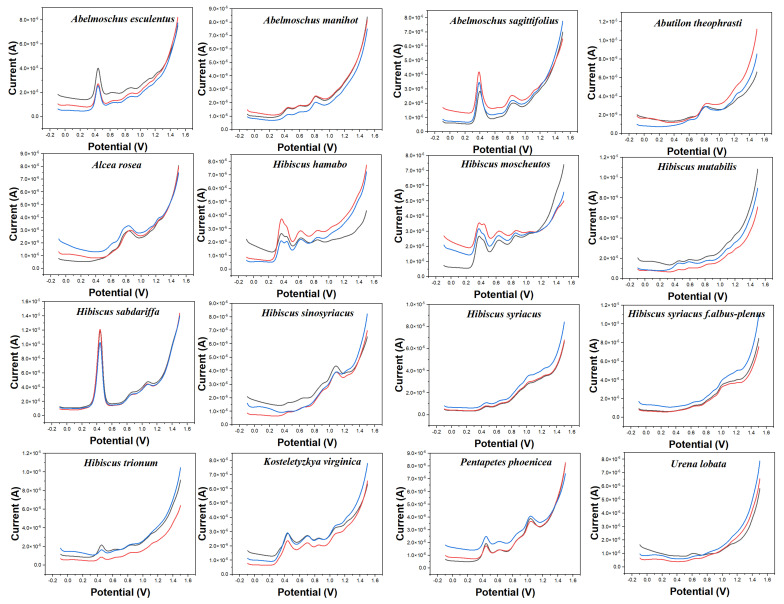
Electrochemical fingerprints of *Abelmoschus esculentus*, *Abelmoschus manihot*, *Abelmoschus sagittifolius*, *Abutilon theophrasti*, *Alcea rosea*, *Hibiscus hamabo*, *Hibiscus moscheutos*, *Hibiscus mutabilis*, *Hibiscus sabdariffa*, *Hibiscus sinosyriacus*, *Hibiscus syriacus*, *Hibiscus syriacus* f. *albus−plenus*, *Hibiscus trionum*, *Kosteletyzkya virginica*, *Pentapetes phoenicea*, and *Urena lobata* recorded after ethanol extraction under 0.1 M ABS (pH 4.5).

Figure 2 shows the electrochemical behavior of the leaves of 16 species collected in this work in 0.1 M ABS after ethanol extraction. The reason to change the buffer solution is to fully demonstrate the difference in the electrochemical behavior of different electrochemically active substances in different pH environments. According to our previous experience [17,20,22,24,41], electrochemically active small molecules under acidic conditions tend to exhibit high signal abundance in the scanned potential interval. In addition, changing the solvent can make a difference in the molecules being extracted. More abundant signals of electrochemically active substances in plant tissues can be obtained by combining fingerprints under different conditions. The background of the electrode in ABS is shown in Figure S2. Similarly, the bare electrode only showed an increase of current at high overpotential without an obvious electrochemical oxidation peak. The electrochemical behavior of each species in Figure 2 is different from those in Figure 1. For example, *Abelmoschus manihot* only shows a large oxidation peak at about 0.60 V in PBS, but it shows three consecutive electrochemical oxidation peaks at 0.42 V, 0.60 V, and 0.80 V, respectively, in ABS. Similarly, *Alcea rosea* has only a gentle oxidation peak in PBS, but its electrochemical fingerprint in ABS contains three distinct characteristic peaks. In addition to a large oxidation peak at 0.85 V, it shows two weak oxidation peaks at 0.60 V and 1.21 V. At the same time, the electrochemical behavior of *Alcea rosea* and *Hibiscus sinosyriacus* is also significantly different under the condition of ABS. It is worth noting that a number of species are also included under ABS, which enjoy similar electrochemical behavior. *Hibiscus mutabilis* and *Urena lobata*, for example, both show a continuous series of small oxidation peaks. *Hibiscus hamabo* and *Hibiscus moscheutos* have a fourth distinct oxidation peak. Therefore, the use of electrochemical fingerprinting to identify different species is still a challenge.

**Figure 3:**
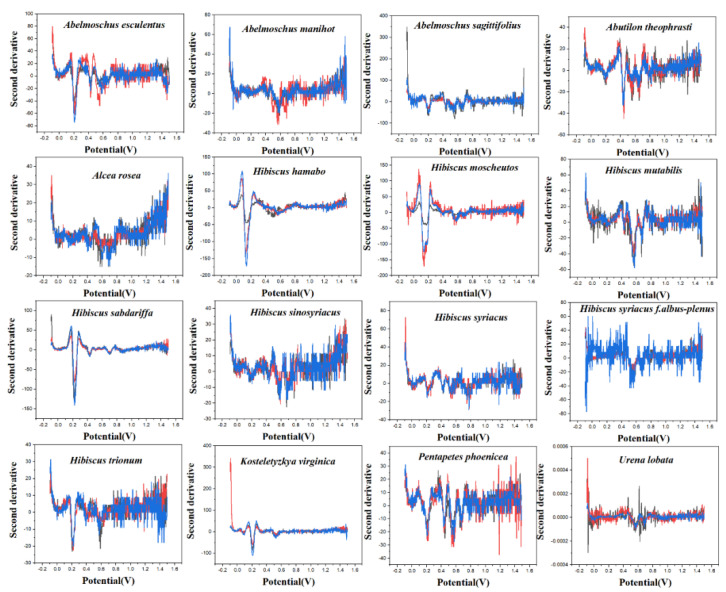
The second derivative curve of electrochemical fingerprint recorded under 0.1 M PBS (pH 7.0).

**Figure 4:**
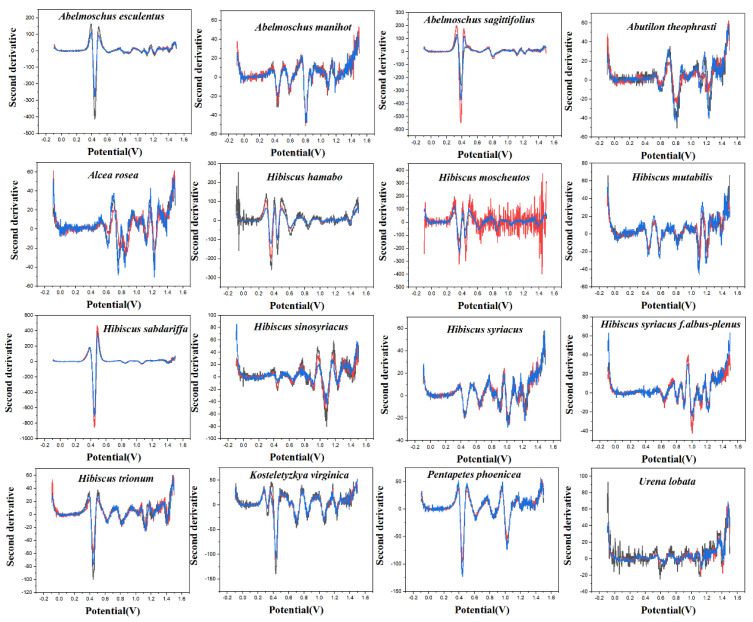
The second derivative curve of electrochemical fingerprint recorded under 0.1 M ABS (pH 4.5).

PLS-DA converts the data from the training set into the intermediate potential variables used to predict the validation set class [42]. Because an appropriate number of potential variables can fully describe the data, in order to best distinguish samples of different categories, the ten-fold cross-validation method is used to obtain the best number of potential variables. Too many potential variables would make it impossible to fit the model, so the number of potential variables in this study was limited to 15. The LinearSVC is an algorithm that uses One-vs-All to implement multiple classifications [43]. It has better performance for models with large amounts of data and is suitable for multi-classification models. The performance of the RF model is mainly evaluated by n. estimators. It represents the number of decision trees, and the number and recognition accuracy generally show a positive correlation, but the stability of the model will decline [44]. The original electrochemical fingerprints of all species and the second derivative electrochemical fingerprints (Figure 3 and Figure 4) were used to learn the three models. In addition, the electrochemical fingerprints of all species collected under PBS and ABS were combined to test the three models. The modeling results are shown in Table 1.

As can be seen from Table 1, the original spectral data after second-derivative processing can significantly improve the accuracy of the model in most cases, especially when ABS + PBS is used as data. These results indicate that the second derivative can significantly reduce the noise in the electrochemical fingerprinting of plant samples, highlight the fingerprint differences of different species, and retain the effective fingerprint information. Among the models established by the three algorithms, the results obtained by ABS + PBS after the second derivative processing have higher accuracy. The prediction set accuracy of the RF optimal model is 85.64%, which is significantly lower than that of the PLS-DA model (96.42%) and the LinearSVC model (97.63%). Among them, the accuracy of the training set in the RF model reaches 100%, while the accuracy of the prediction set is poor, which indicates that the model of this method may be overfitting. Therefore, the ABS + PBS PLS-DA model and the LinearSVC model after second derivative processing are more suitable for the classification and identification of Malvaceae species.

**Figure 5:**
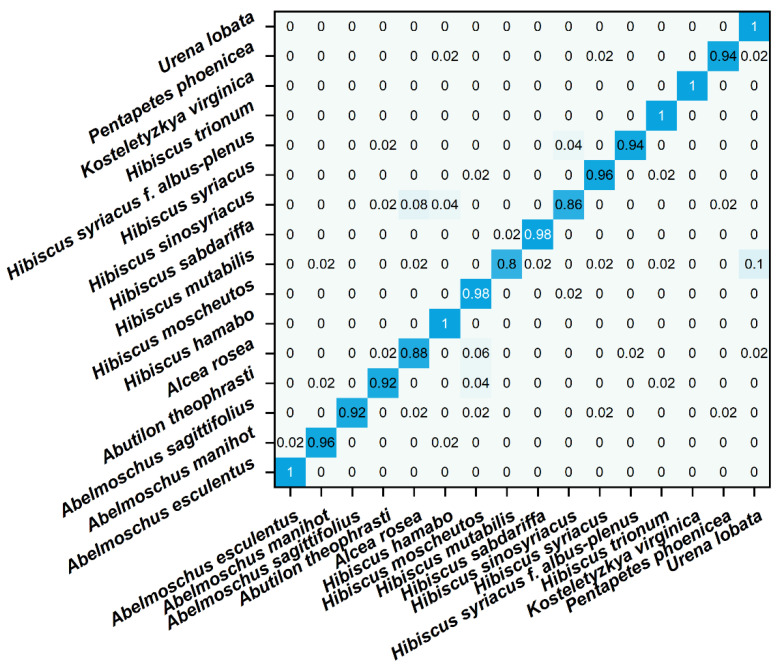
Confusion matrix of identification of plant species using electrochemical fingerprint (ABS + PBS) via LinearSVC.

The obfuscation matrix visualizes the comparison between the predicted value and the true value of the model in matrix form [45]. Each row in the matrix represents the predicted value of a different species, and each column represents the true value of a different species. In the confusion matrix of this experiment, blue indicates the accuracy of recognition. The depth of the color is proportional to the more accurate recognition. Figure 5 shows the confusion matrix of different species identified by the LinearSVC model. It can be seen that most species can be recognized, but *Hibiscus mutabilis* and *Hibiscus sinosyriacus* show a low recognition rate. In future work, other data processing methods need to be tried to optimize the recognition efficiency. At the same time, different models can be tried. Spectral data from plant samples are widely used for species identification, but this technique is not widely used in electrochemical fingerprinting. This may be because electrochemical fingerprinting is a new fingerprint technology developed in recent years. Because the collection of electrochemical signals does not involve the separation of samples, its accuracy is limited. Our work explores the feasibility of applying common classification models to electrochemical fingerprinting. However, electrochemical fingerprints combined with different conditions can be used for identification only if they show the electrochemical behavior of different electrochemically active substances. Although we have used different buffer solutions (different pH) and different solvents to achieve this in this work, it is a priori assumed. In future work, the composition analysis of extracts used for electrochemical fingerprinting is an effective way to verify this hypothesis. The effectiveness of this methodology can be optimized by further regulating the conditions for electrochemical fingerprinting through component analysis.

**Figure 6:**
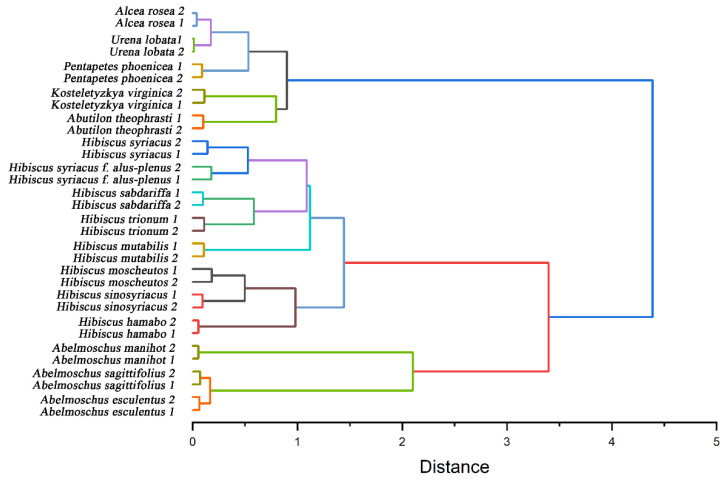
Dendrogram of *Abelmoschus esculentus*, *Abelmoschus manihot*, *Abelmoschus sagittifolius*, *Abutilon theophrasti*, *Alcea rosea*, *Hibiscus hamabo*, *Hibiscus moscheutos*, *Hibiscus mutabilis*, *Hibiscus sabdariffa*, *Hibiscus sinosyriacus*, *Hibiscus syriacus*, *Hibiscus syriacus* f. *albus-plenus*, *Hibiscus trionum*, *Kosteletyzkya virginica*, *Pentapetes phoenicea*, and *Urena lobata* based on electrochemical fingerprints.

Further, we used the electrochemical fingerprint data to cluster these species for phylogenetic analysis. Figure 6 shows a dendrogram based on the electrochemical fingerprints of all species of leaves collected under PBS and ABS. Two independent samples of each species participate in the clustering. All the species are divided into three clusters. The first cluster included *Abelmoschus esculentus*, *Abelmoschus manihot*, and *Abelmoschus sagittifolius*. The locations of three species in the *Abelmoschus’* cluster are consistent with results reported in recent years from complete chloroplast genomes [46]. The second cluster includes *Hibiscus hamabo*, *Hibiscus moscheutos*, *Hibiscus mutabilis*, *Hibiscus sabdariffa*, *Hibiscus sinosyriacus*, *Hibiscus syriacus*, *Hibiscus syriacus* f. *albus-plenus*, and *Hibiscus trionum*. *Hibiscus* species are also clustered together, but their relationships are not consistent with morphological classification results. For example, morphological results show that *Hibiscus hamabo* and *Hibiscus moscheutos* are closely related [47]. Meanwhile, the relationship between *Hibiscus mutabilis* and *Hibiscus sinosyriacus* is relatively close. However, the relationship between *Hibiscus hamabo*, *Hibiscus sinosyriacus*, and *Hibiscus moscheutos* in our results is relatively close. *Hibiscus mutabilis* is in another cluster. Both clusters bring together species from the same genus. This well represents that the information collected by electrochemical fingerprint sensing technology can distinguish the differences of different plants at the genetic level. This is because the electrochemical fingerprint signals reflect the differences between the electrochemically active substances in the plant tissue. The differences of electrochemically active substances can be further used to reflect the differences at the genetic level between different species. Species within the same genus will have less genetic variation than species within different genera.

## 4. Conclusions

Electrochemical fingerprinting was used to collect the electrochemically active substances in Malvaceae leaves. Different species exhibit different electrochemical behaviors. The same species showed a steady signal. These fingerprint signals can be more effectively used for species identification by second derivative processing. PLS-DA, LinearSVC, and RF were used to identify the original electrochemical fingerprint signal and the processed signal. The results show that LinearSVC has the best identification efficiency for the second derivative processing of electrochemical signals (ABS + PBS). Electrochemical fingerprint signals are further used to investigate plant phylogeny. The results show that 18 species can be divided into three clusters. Among them, all species of Hibiscus and Abelmoschus were gathered together, indicating that the electrochemical fingerprint signal can reflect the genetic differences between different species. In addition, the results of phylogenetic surveys are compared with those of other taxonomic techniques.

## Figures and Tables

**Table 1:** Identification accuracy of electrochemical fingerprints and its second derivative data using PLS-DA, LinearSVC, and RF.

Algorithm of Classification	Data Treatment	ABS		PBS		ABS + PBS	
		Training Set	Prediction Set	Training Set	Prediction Set	Training Set	Prediction Set
PLS-DA	N/A	92.40	88.05	88.01	85.20	90.54	88.15
	Second derivative	95.51	93.63	96.57	91.70	98.51	96.42
LinearSVC	N/A	93.20	91.24	91.27	89.80	92.24	91.52
	Second derivative	97.22	95.41	98.20	95.42	99.52	97.63
RF	N/A	100.00	76.52	100.00	77.75	100.00	81.25
	Second derivative	100.00	81.71	100.00	79.62	100.00	85.64

## Data Availability

Data sharing not applicable.

## Supplementary Materials

The following supporting information can be downloaded at: https://www.mdpi.com/article/10.3390/mi14020248/s1.
