# Supplementary material for: Analysis of Electrochemically Active Substances in Malvaceae Leaves via Electroanalytical Sensing Technology for Species Identification
Source: Micromachines (Basel). 2023 Jan 18;14(2):248. doi: 10.3390/mi14020248 (PMC9963770; doi:10.3390/mi14020248)
Supplement: Supplementary file 1 [file micromachines-14-00248-s001.zip › micromachines-2141775-supplementary.pdf]

micromachines

Supplementary Materials

Analysis of Electrochemically Active Substances in Malvaceae Leaves Via Electroanalytical Sensing Technology for Species Identification

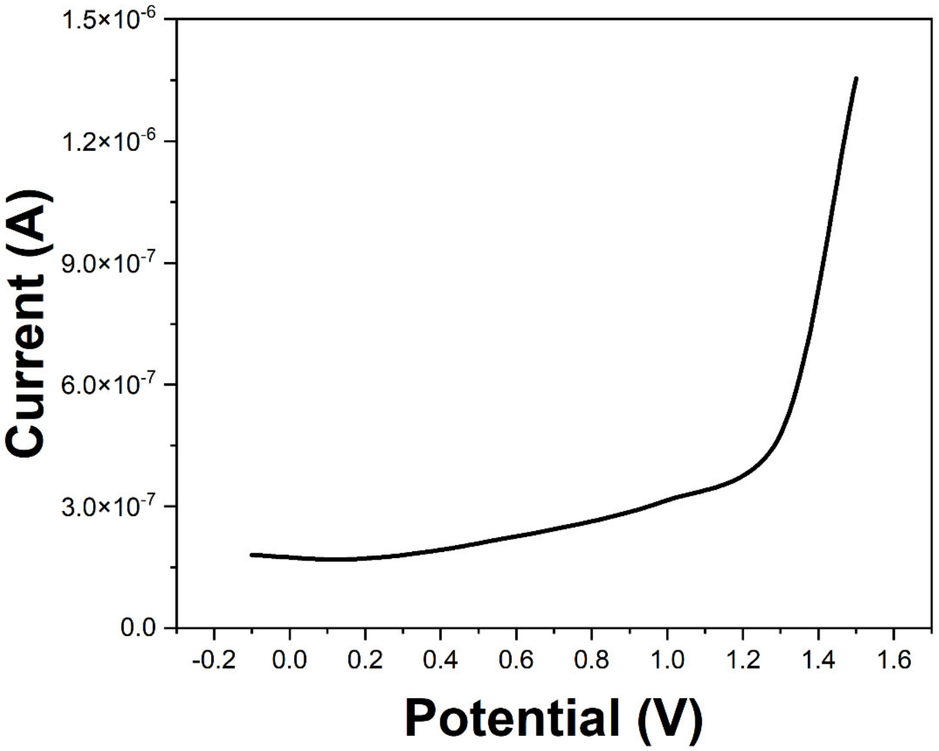

Figure S1. DPV curve of bare glassy carbon electrode in 0.1 M PBS (pH 7.0).

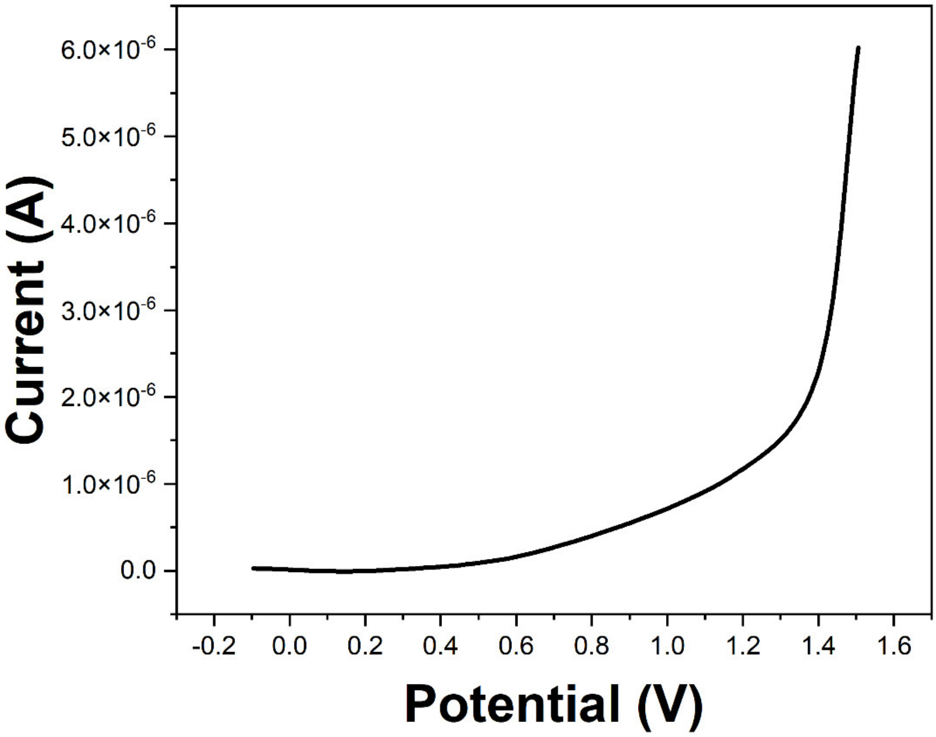

Figure S2. DPV curve of bare glassy carbon electrode in 0.1 M PBS (pH 7.0).

Supporting Information S1: Extraction Process

All extraction process was conducted at 25°C. Water and ethanol were used as the solvents in the extraction procedure. Specifically, 0.3 g leaves were cut and added to 5 mL of solvent. The mixture was supplemented with four grinding beads. The tube was put in a tissue grinding device (Meibi-96, Zhejiang, China) for 2 min extraction under 80 Hz. After waiting for precipitation, the supernatant was collected for electrochemical fingerprint collection.

Supporting Information S2: Electrochemical Fingerprints Collection

Phosphate buffer solution (PBS, 0.1 M, pH 7.0) and acetic acid buffer (ABS, 0.1 M, pH 4.5) were used as electrolytes to support electrochemical fingerprint collection. Electrochemical fingerprinting was determined using a traditional three-electrode system. A glassy carbon electrode, a platinum wire and an Ag/AgCl electrode were used as working electrode, counter electrode and reference electrode, respectively. All electrochemical experiments were conducted under a CHI 760E working station at room temperature. A differential pulse voltammetry (DPV) was recorded from −0.1 to 1.5 V (pulse amplitude: 50 mV; pulse width: 0.05 s; pulse period: 0.5 s). The experimental data was then normalized for further analysis.
